# Ligand-binding specificity and promiscuity of the main lignocellulolytic enzyme families as revealed by active-site architecture analysis

**DOI:** 10.1038/srep23605

**Published:** 2016-03-24

**Authors:** Li Tian, Shijia Liu, Shuai Wang, Lushan Wang

**Affiliations:** 1 The State Key Laboratory of Microbial Technology, Shandong University, Jinan, 250100, P.R. China; 2 Taishan College, Shandong University, Jinan, 250100, P.R. China

## Abstract

Biomass can be converted into sugars by a series of lignocellulolytic enzymes, which belong to the glycoside hydrolase (GH) families summarized in CAZy databases. Here, using a structural bioinformatics method, we analyzed the active site architecture of the main lignocellulolytic enzyme families. The aromatic amino acids Trp/Tyr and polar amino acids Glu/Asp/Asn/Gln/Arg occurred at higher frequencies in the active site architecture than in the whole enzyme structure. And the number of potential subsites was significantly different among different families. In the cellulase and xylanase families, the conserved amino acids in the active site architecture were mostly found at the −2 to +1 subsites, while in β-glucosidase they were mainly concentrated at the −1 subsite. Families with more conserved binding amino acid residues displayed strong selectivity for their ligands, while those with fewer conserved binding amino acid residues often exhibited promiscuity when recognizing ligands. Enzymes with different activities also tended to bind different hydroxyl oxygen atoms on the ligand. These results may help us to better understand the common and unique structural bases of enzyme-ligand recognition from different families and provide a theoretical basis for the functional evolution and rational design of major lignocellulolytic enzymes.

Lignocellulose is mainly composed of cellulose, hemicellulose and lignin^1^, with high heterogeneity of the polysaccharide constituents as previously proposed^2^. The efficient and complete degradation of lignocellulose is the most important step in the global carbon cycle^3^, and it is also a major obstacle to the large-scale utilization of biomass resources to produce new types of energy^4^,^5^. Hence, it is crucial to understand this degradation process, due to the heterogeneity of lignocellulose, which requires a variety of enzymes to act synergistically^6^. Such diversity makes it more difficult to understand the degradation process in detail.

With the rapid development of sequencing technology, biological technology has entered the era of big data, which makes it possible to uncover regularity through the fast and in-depth analysis of massive sequences. Therefore, it is necessary to develop new methods employing computational tools to extract sequence information^7^,^8^. Such information can generate “small but smart libraries” to aid the understanding of function or guide experiments, greatly reducing the labor and time needed^9^,^10^. Among the databases containing relevant sequence information, the CAZy database is a knowledge-based resource specializing in enzymes that synthesize and degrade complex carbohydrates and glycoconjugates. CAZymes are classified into several distinct families based on amino-acid sequence similarity^11^,^12^. The lignocellulose degradation enzymes are classified under the category of glycoside hydrolase^13^, which included 135 families by October 2015. Within each family, members display conserved topology structures and catalytic characteristics^14^,^15^.

As the criteria for classifying different enzyme families in the CAZy database do not include ligand specificity, there may be multiple functions within a single GH family. For example, GH5 contains members with cellulase, xylanase and β-glucosidase activities. Functional promiscuity within families is very common^16^, and partial recognition between enzymes and their ligands is the most likely mechanism to cause promiscuity^17^. Research into the structural basis of recognition has become a promising field as scientists seek to further understand the reasons underlying this phenomenon.

Studies have shown that there exists a level of protein structure different from the traditional hierarchy: sectors^18^, which contain a few amino acids that determine the biological function of the protein. The specificity of these amino acid forms gradually during evolution, which also explains why sectors of enzymes within a family characterized by promiscuity are different^19^. In terms of enzymes, one of the most important sectors is referred to here as “active site architecture”^20^, which is the combination of amino acid residues that make direct contact with the ligand and perform enzymatic functions. Its distribution area is known as the active region, accounting for about 2%–3% of the whole enzyme and it is affected by the length of ligand: an enzyme with a longer ligand has larger active sites^21^,^22^. Analyses focusing on the active site architecture can reveal a substantial amount of information and therefore have been used in many previous studies, such as those investigating the structural features of the ligand binding site of galactose-binding proteins^23^ and protein kinase subfamily specific sites^8^. In addition, this approach has also been applied to the GH families, for example in the work by Kumar, which analyzed the key amino acid residues and space conservation of the GH13 family amylase active region^24^,^25^,^26^, and that by Chen, which revealed the motif that determines glucan and mannan double ligand specificity in the GH5-4 subfamily through phylogenetic analysis^27^, and Liu *et al.* revealed the ligand-binding specificity of chitinase and chitosanase by active-site architecture analysis^28^.

This paper applied similar analyses to nine GH families. Among all of the components in lignocellulose, only cellulose and hemicellulose can be converted into fermentable sugars using microbial cellulase and hemicellulase^1^, which include a variety of enzymes. Based on ligand specificity, three representative enzymes were selected, cellulase, xylanase and β-glucosidase, and statistical analyses of the biological information on their active site architectures were performed. The results illustrate the differences between different enzymes during the process of ligand recognition, and offer a possible explanation for the functional promiscuity seen within certain enzyme families. Moreover, these findings could also aid in understanding the complex process of enzyme-ligand interactions at the molecular level.

## Results and Discussion

### Amino acid frequencies and preferences in the active site architecture

All sequences of selected lignocellulolytic enzyme families were obtained from CAZy database. There were three enzyme classes, cellulase, xylanase and β-glucosidase. GH5, GH6, GH7, GH9 and GH12 were selected as the target families of cellulase with 522, 63, 82, 157 and 65 sequences, respectively; GH10 had 335 sequences and GH11 had 267 sequences to represent xylanase; GH1 and GH3 contained 331 and 278 sequences to represent β-glucosidase (Supplementary Table S1, all data above was valid to October 2015). All sequences were characterized as specific activities and used for later analysis.

**Figure 1:**
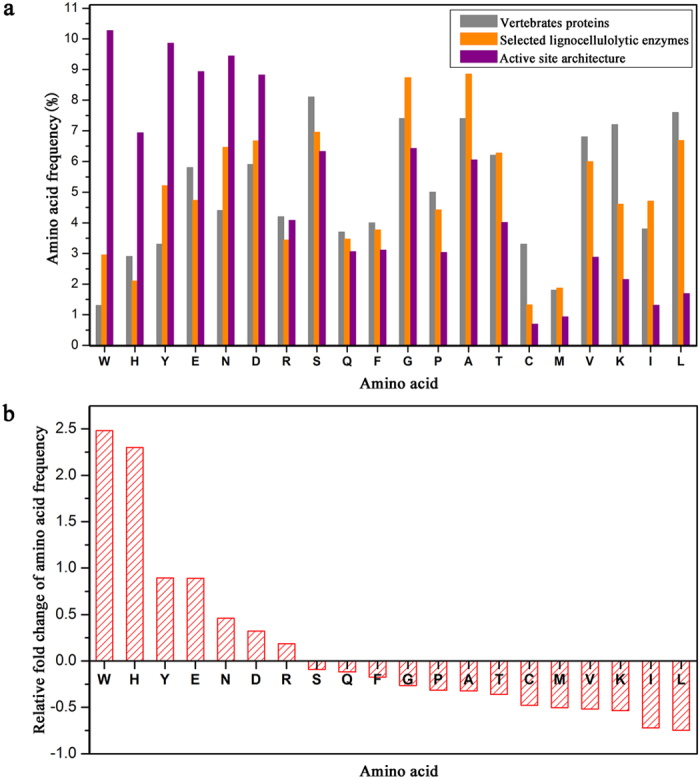
Comparison of amino acid frequencies. (**a**) Amino acid frequencies of vertebrate proteins and selected lignocellulolytic enzymes and their active site architectures. The PEARSON correlation coefficient (r) of the first two sets was 0.80419 (the correlation is extremely strong when r > 0.8), indicating that the elements contained in them were similar; the correlation coefficient of the last two sets was 0.29385 (two groups of data are weakly related when 0.2 < r < 0.4), thus no significant difference was observed. (**b**) Relative fold changes of amino acid frequencies between selected lignocellulolytic enzymes and their active site architectures. Positive-, zero-, and negative-fold changes indicate that amino acid frequencies within the active sites were higher than, equal to, or lower than those observed in the whole enzyme, respectively.

The frequencies of 20 amino acids in vertebrate proteins^29^, selected lignocellulolytic enzymes and their active site architectures determined by the combination of amino acids within 5 Å of the ligand were calculated (Fig. 1a). The results showed that the frequencies of the 20 amino acids occurring in vertebrate proteins and lignocellulolytic enzymes were strongly correlated (r = 0.80419), consistent with the report that the amino acid frequencies within proteins are the result of the random arrangement of the genetic code^29^. However, the frequencies of the 20 amino acids present in the active site architecture of lignocellulolytic enzymes were remarkably different from those of the whole lignocellulolytic enzymes (r = 0.29385). Together with the relative fold change further calculated (Fig. 1b), it can be seen that, compared with the overall enzyme, certain amino acids occur preferentially in the active site architecture.

Seven amino acids (Trp, His, Tyr, Glu, Asp, Asn and Arg) demonstrated elevated frequencies in the active site (Fig. 1b). These amino acids can be divided into two types according to their properties: hydrophobic and polar. Protein folding in aqueous media always tends to bury hydrophobic amino acid inside the molecule, and the stability of the protein tertiary structure is maintained by hydrophobic interactions^30^. Protein-ligand interactions mainly conducted by amino acids on the protein interface, so there are fewer hydrophobic amino acids in the active site. This agrees with the observation that the frequencies of hydrophobic amino acids such as Leu, Ile, Val, Met, Ala, Pro and Phe in the active site were reduced by 75%, 72%, 52%, 50%, 32%, 31% and 18%, respectively, compared with those in the whole enzyme. However, the interesting result was that the frequencies of Trp and Tyr in the active site were higher by 248% and 89%, respectively, compared with the whole enzyme, especially Trp has the highest frequency in the active site, indicating the important roles it plays in enzymatic function. This result was in accordance with the previous report, which stated that the aromatic amino acid residues line the active site of the GHs from the observation of structure^31^,^32^,^33^, and these amino acid residues were proved to participate in the binding and stabilization of carbohydrate ligands by molecular dynamics simulations and biochemical experiments^34^,^35^,^36^. In addition, the polar amino acids, His, Glu, Asp, Asn and Arg, appeared more often in active sites, with their frequencies increased by 230%, 89%, 46%, 32% and 19%, respectively, while Lys and Thr occurred less often. Similarly, those amino acid residues also played significant roles in ligand binding and catalysis^37^,^38^. The analysis above suggests that the active site amino acid residues of lignocellulolytic enzymes are not random. The preferred amino acid residues gather at the active site gradually to form a functional area under the pressure of natural selection^39^, which is also the structural bases of enzymatic function.

### Statistical analysis of amino acid residue numbers at potential subsites

In order to accurately assess the affinity between the enzyme and ligand, it was proposed that the ligand-binding region of a glycoside hydrolase can be recognized as an array of tandem arranged subsites, where each subsite contains amino acid residues interacting with a single glycosyl unit of ligand^40^,^41^. It is well recognized that members of a certain CAZy family have a similar 3D topological structure, and the number and location distributions of potential subsites are similar. However, different families may have different sizes of catalytic clefts or tunnels due to the different topological structures (Supplementary Table S2). Considering that the size of the pyranose or furanose ring is 5.2 Å, the number of potential subsites is also proportional to the length of the cleft or tunnel (Supplementary Fig. S1). Here, the number and location distributions of potential subsites were different in different families as shown in Supplementary Figs S2–S4. The number of subsites ranged from 2 to 9, and the distribution of subsite locations ranged from −7 to +4. For each family, the number of amino acid residues at each subsite was then calculated in Tables 1, 2, 3. Interestingly, the distribution of amino acid residue density was not symmetrical to the cleavage position: higher amino acid residue densities occurred at the non-reducing end, while lower densities were found at the reducing end. The asymmetry in this structural basis is likely to account for the different modes of action of ligand binding and product release. However, comparing enzymes that exhibit the same activity but belong to different families, the average number of amino acid residues at a single subsite was relatively stable, with cellulase having 4.4 ± 0.7 amino acid residues (the plus/minus referring to standard deviations, and 889 sequences were used to for analysis), xylanase having 3.4 ± 0.5 (602 sequences were adopted) and β-glucosidase having 7.0 ± 0.0 (609 sequences were adopted) (Tables 1, 2, 3) . This suggests that enzymes such as β-glucosidase that have fewer ligand binding subsites are characterized by larger average numbers of amino acid residues per subsite, which ensures a certain amount of total subsite-ligand binding energy.

There were also subsites common to different families that exhibit the same activity, and the average numbers of amino acid residues found at each common subsite were also calculated (Tables 1, 2, 3) and compared with the average number of amino acid residues at all subsites. In cellulase and xylanase there were three subsites, −2, −1 and +1, that had more amino acid residues than the total average value, whereas in β-glucosidase only the −1 subsite displayed this pattern. This suggests that, when degrading ligands with long or short chains, each enzyme has its own main subsite(s). But the above-mentioned subsites with higher binding energies primarily account for ligand binding and catalysis, while other subsites play a supporting role. In other words, interactions with the −2 to +1 subsites are indispensable for ligand binding, stabilization and catalysis for cellulase and xylanase, which means the smallest structural unit necessary for enzyme binding is at least a triose^13^, and this is the only explanation for the essential existence of subsites −2, −1 and +1. Hence, the amino acid residues at those three subsites have become the focus when rationally designing new enzymes. It has been reported that different cellulases have different action modes^42^, and the transform of action mode may be achieved by mutating amino acid residues at −2 to +1 subsite. For example, *Tr*Cel12A (GH12), the minimum binding unit of wild type is cellotetraose, while the mutant (W22Y at −2 subsite) can bind and degrade cellotriose through increasing the binding affinity at −2 subsite^43^. Similarly, it can also be applied to alter the action mode of xylanase and other glycoside hydrolases through increasing or decreasing the binding affinity.

In addition to the common patterns above, each family had its own characteristics. In the cellulase families, there were 35 amino acid residues in total at potential subsites in GH6, GH7 had 48 amino acid residues (Table 1), and these two families had many more amino acid residues than the other three families (GH5, GH9 and GH12 with 19, 26 and 23 amino acid residues, respectively). In general, the ligand-enzyme binding energy increase with increasing numbers of amino acid residues, so GH6 and GH7 enzymes have the higher binding energies. In addition to the high binding energy is the structural basis for evolving an exoglucanase, and this is also the reason why exoglucanase activity mostly exists in GH6 and GH7. However, a higher binding energy does not necessarily coincide with higher catalytic efficiency, for it has been observed that CBHI (GH7) exhibits a significantly slower catalytic rate compared to EGIII (GH12)^44^. And this phenomenon can be found in a wide range of relevant research work, for example, reduced binding energy between GH7 enzymes and ligand could result in enhanced hydrolytic activity^45^; higher binding energy between cellohexaose and Man5B (GH5) inhibited enzymatic activity^46^; improving the binding energy of *Tr*Cel12A (GH12) at different subsites resulted in distinct effects (either increased or reduced) to enzymatic activity. Therefore, higher catalytic efficiency requires a good balance between ligand binding and product release^46^.

### Sequence profile characteristics of the active site architecture

**Figure 2:**
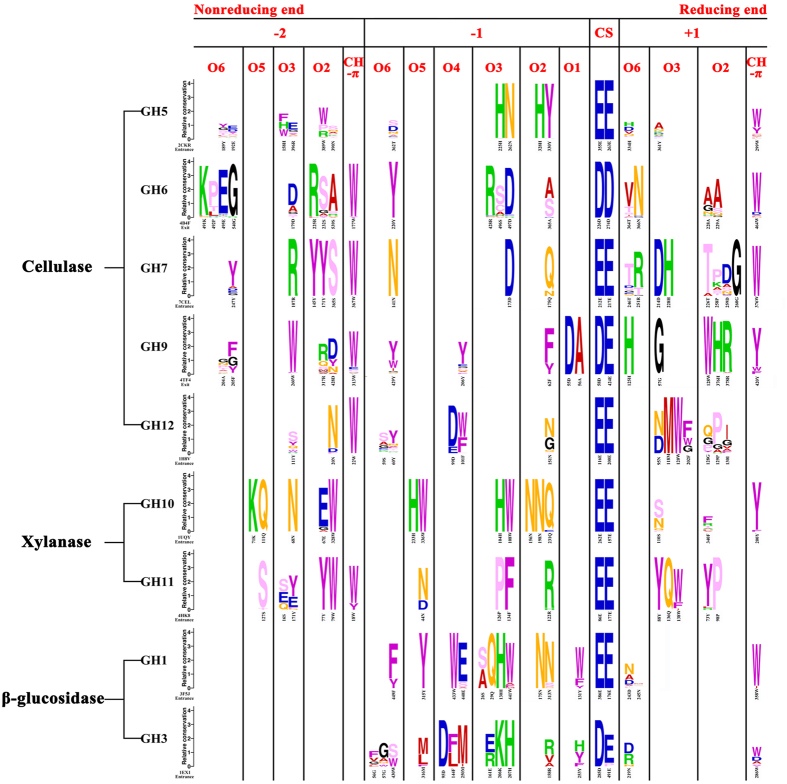
Sequence profiles of different lignocellulolytic enzyme families. In each sequence profile of a certain family, the ordinate indicates the relative degree of conservation, while the abscissa represents PDB ID of the template structure, as well as types and serial numbers of each amino acid. Each type of amino acid is represented by abbreviated letters with a corresponding color (KRH: green; DE: blue; MVALI: red; G: black; FWY: purple; NQ: RGB = FFB300; TSPC: RGB = FFB3FF), where the same color suggests a similar property of the amino acid. In each row of sequence profiles, the heights of all letters represent the relative degrees of conservation, while the height of a single letter denotes its specific occurrence frequency. The locations of ligand atoms interacting with the amino acid residues at each subsite are marked at the top, where “CS” stands for “cleavage site”.

Previous studies^42^,^43^ showed that in the active site architecture, the amino acid residues at −2 to +1 subsites contain information that be helpful to understand the action mode of an enzyme. Thus, the sequence profiles of −2 to +1 subsites would be demonstrated in Fig. 2 with further analysis. Many catalytic characteristics are common to these nine families. The catalytic mechanism is either retaining or inverting; Glu and/or Asp are located at the cleavage site to perform catalysis, and are strictly conserved within each of the selected families except for GH3. Moreover, extending from the catalytic amino acid residues in both directions, there are many other amino acid residues exhibiting a high degree of conservation, such as 262N, 328H and 330Y in 2CKR (Protein Data Bank Identification, PDB ID of template structure), 220Y in 4B4F and 71 K and 111Q in 1UQY. These conserved amino acid residues have been sustained during evolution, but their types and distributions among different families are significantly different, which can be seen clearly from Fig. 2.

It has been reported that the CH-π interaction, mainly contributed by the aromatic amino acids Trp, Tyr and Phe, plays an important role in the protein-carbohydrate recognition process^47^. As shown in Fig. 2, there are no CH-π interactions in the −1 subsites in any of the families, but this interaction does occur more frequently in the −2 or +1 subsites. Through these short-range CH-π interactions, the aromatic amino acids align with the pyranose rings at one or both sides of the cleavage site, which possibly guarantees the precise positioning of the carbohydrate chain to aid the accuracy of the hydrolytic process. Because their mode of action requires them to lie parallel to the pyranose ring, these aromatic amino acids can be called “stable amino acid residues”. In addition, distortion of the pyranose ring at the −1 subsite is necessary to break the glycosidic bond^48^,^49^, and stabilization of this conformational change is achieved by amino acid residues at this subsite. Most amino acid residues occurring at −1 subsites are polar, such as His, Asp, Asn, Gln and Arg, which are able to form hydrogen bonds with hydroxyl groups on the ligand. However, the types of hydroxyl groups the amino acid residues act on changes according to the family (Fig. 2), which means the position and intensity of the hydrogen bond varies as well as the distinct degree of torsion of the pyranose ring, which may be one of the reasons for diverse manners of bond-breaking in different families^50^.

In general, amino acid residues frequently appearing on the sequence profile are polar amino acid residues and aromatic amino acid residues, and they interact with the ligand by either hydrogen bonding or hydrophobic interactions. Among the cellulase families, there are almost no Arg and Lys amino acid residues except for GH6 and GH7 (GH6 has two Arg and one Lys, GH7 has five Arg and one Lys, Supplementary Fig. S2). Interestingly, these two families contain most of the exoglucanase that can degrade crystalline cellulose, which exhibits a higher binding energy. So it is reasonable to speculate that Arg and Lys are associated with higher binding energy. In terms of the xylanase families, the number of polar amino acid residues in GH10 was almost twice those in GH11, whereas the distribution of aromatic amino acid residues was just the opposite, illustrating that GH10 and GH11 might have different preferences for hydrogen bonding versus hydrophobic interactions. Hydrogen bonding is more accurate than hydropholic interaction during the process of enzyme-ligand interaction, which leading to slower ligand binding or product release. Since the catalytic efficiency is decided by the both of them, it is reasonable that GH10 exhibits a significantly slower catalytic efficiency compared to GH11^51^. And compared with GH1 β-glucosidase, the amino acid residues in GH3 had a lower degree of conservation, and this shows there might be more diverse binding modes and functional promiscuity in GH3.

In order to verify the reproducibility of our results, we adopted another two methods to verify the accuracy of conserved amino acid residues in the sequence profile as shown in Supplementary Figs S2–S4, and the results well supported our consistent observation of the degree of conservation (Supplementary Table S3). In the past few years, the available sequences have increased rapidly in the CAZy database, while enzymatic function is not growing significantly^11^, such as GH5, GH10 and GH11 families. We believe this trend will continue in the coming years. Therefore, conserved amino acid residues obtained using Weblogo were intrinsic, and they remained considerably unchanged during the evolution process, maintaining conserved structures and functions of the enzymes, whose function may be partially or completely lost if part of the sequence is mutated.

### Ligand-binding specificity and functional promiscuity of different families

**Figure 3:**
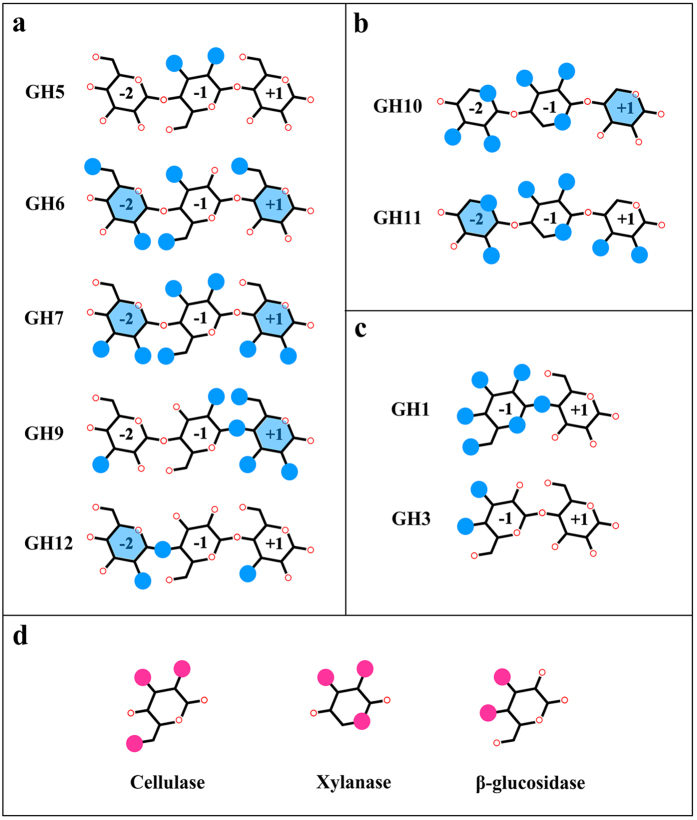
Specific modes of interaction of conserved amino acid residues with ligands in selected families. (**a**) Cellulase families; (**b**) Xylanase families; (**c**) β-glucosidase families; (**d**) Overall portrait of the three enzyme families. In (**a**–**c**), the oxygen atoms that the conserved amino acid residues interact with are highlighted with blue circles, and the blue transparent hexagon on the pyranose ring represents the CH-π interaction. In (**d**), the oxygen atoms at all sites are displayed in the same pyranose ring, and the large pink circle denotes oxygen atoms recognized by different families with the same enzyme activity. If amino acids of the same type, or with similar properties, make up more than 90% of the sequences in any column of multiple sequence alignment (that is, amino acids of a single color make up the whole column in the sequence profile), this column is defined as conserved.

Figure 3 clearly shows the oxygen atoms on the ligands that interact with the conserved amino acid residues (but not the catalytic residues), and the space positions of ligands and amino acid residues are displayed in Supplementary Fig. S5. As is well known, cellulose is composed of glucose monomers connected by β-1,4 glycosidic bonds. Considering the six oxygen atoms of the glucose ring, the hydroxyl oxygens on C1, C2, C3, C4 and C6 are denoted O1, O2, O3, O4 and O6, respectively, while the oxygen on the pyranose ring is denoted O5. Similar nomenclature was also adopted for xylose, apart from the absence of the hydroxyl group on C6.

As shown in Fig. 3a, the conserved amino acid residues interact only with O2 and O3 at the –1 site in members of the GH5 family, and they comprise a smaller proportion of all interacting amino acid residues compared with the other cellulase families. This is in accordance with the existence of the subfamilies in GH5, demonstrating that they have various ligand specificities. Selected enzymes of GH6 have conserved amino acid residues binding to O2 and O6 at the −2 site, O3 and O6 at the −1 site and O6 at the +1 site, while the conserved Trp forms CH-π interactions at both −2 and +1 sites. In members of GH7, the conserved amino acid residues bind to O2 and O3 at all sites and O6 at the −1 site; besides CH-π interactions also exist at the −2 and +1 sites. This illustrates that CH-π interactions play an important role in exoglucanase activity, and conserved amino acid residues are uniformly distributed across all subsites of GH6 and GH7, precluding the existence of protruding functional groups. Additionally, the results also confirm the structural characteristic that the architecture of the exoglucanase active site forms a tunnel. The conserved amino acid residues of GH9 enzymes mainly interact with the +1 site, which suggests that the pyranose rings at the +1 site do not have side chains. However, O6 did not display any interactions with conserved amino acid residues at any sites in GH12, showing the low recognition of the hydroxyl at this specific site. This means that GH12 enzymes possibly can degrade xyloglucan, which has xylose at C6.

In addition, in the xylanase families (Fig. 3b), the conserved amino acid residues were absent at the +1 site in GH10 members, consistent with the report that side-chain modifications of xylan are permitted at this site^52^. Similarly to GH11, O3 at the −2 site can have an arabinofuranosyl substituent^51^. In terms of the two β-glucosidase families, enzymes in GH3 exhibit fewer conserved amino acid residues at the −1 site compared with GH1 (Fig. 3c), which is consistent with the greater diversity of their ligands^53^. Moreover, GH3 is similar with GH5, both of them have significantly less conserved amino acid residues interact with hydroxyl oxygens, so they exhibited promiscuity when recognizing various ligands.

Different enzyme families exhibit different interaction patterns between conserved amino acid residues and ligand oxygen atoms, but ligands with similar structures can be partially recognized by different enzymes, which is the structural basis of a single enzyme having multiple ligands. And it is worth noting that the CH-π interaction has no effect on enzyme-ligand specific recognition, for it is determined by the relative electron density rather than the configuration of any specific atom. In comparison, a hydrogen bond, formed between two specific atoms, is determined by the direction and angle between them, and this also accounts for the high specificity between enzyme and ligand. From what has been discussed above, it is reasonable to infer that the specific enzyme-ligand recognition will be weaker when it mainly depends on the CH-π interaction.

Additionally, we found that the conserved amino acid residues form hydrogen bonds with O2, O3 or O6 in cellulase families but have no effect on O5 (Fig. 3d), although the conserved amino acid residues of xylanase families interact with O2, O3 and O5. This phenomenon arises because of the main structural difference between glucose and xylose, and the presence of an additional –CH_2_OH group at the C5 position in glucose. Hence, it is speculated that xylan can easily enter the active channel of a cellulase, but does not bind strongly. However, the cellulose chain cannot enter the active channel of a xylanase due to steric hindrance at the O6 position. Therefore, the transformation between cellulase and xylanase can be realized by mutating some amino acid residues in cellulase or xylanase. It has been reported that *Ct*Cel5E is a bifunctional enzyme with both cellulase and xylanase activities^54^. A series of mutations have been conducted on this enzyme, and the results showed that it loses both activities if the mutations occur at amino acid residues that have interactions with O2 and O3, for these two hydroxyls are the common structural basis of the recognition of cellulose and xylan by the enzyme. Besides, among these mutations, there has been no report of loss of one enzymatic activity completely without affecting the other one. One probable reason is that this enzyme has no amino acid residues that only interact with O6 or O5, thus the bifunctionality cannot be removed.

β-glucosidase plays an essential role in the cleavage of non-reducing terminal glucosyl groups from saccharides and glycosides. Thus, it must have amino acid residues that specifically bind to the oxygen atoms at the −1 site. Under this condition, conserved amino acid residues mainly act on O3 and O4 located on one side of the pyranose ring (Fig. 3d), which is possibly due to steric hindrance. As a result, the transformation between cellulase and β-glucosidase would be very difficult, because there are too few positions recognized by both of them. So, nature has evolved at least two enzyme systems to completely degrade cellulose by collaborating with each other. According to the common and different structural bases of the ligands, rules by which different enzymes perform their functions have been proposed in this paper, providing not only a means of predicting the presence of bifunctional enzymes, but also a theoretical basis for the transformation between the key enzymes.

In conclusion, we used a structural bioinformatics method to analyze the active site architectures of nine major lignocellulolytic enzyme families. The data show that the frequencies of occurrence of amino acids in the active site architecture display obvious preferences, with more frequent appearances of Trp, His, Tyr, Glu, Asp, Asn and Arg. By analyzing the sequence profiles of active site architectures, the structural basis of ligand recognition for each selected family was identified. Furthermore, some relevant conclusions were put forward, such as the importance of the −2 to +1 subsites in the cellulase families, the proportions of aromatic amino acid residues and polar amino acid residues in xylanase families and the specific recognition of the −1 subsite in β-glucosidase families. By locating the specific ligand atoms that the conserved amino acid residues bind to, the specific and promiscuous pattern of ligand binding in each family was analyzed, and the functional differences among the different enzyme families were further revealed. These findings could ultimately provide a theoretical basis for the rational design of lignocellulolytic enzymes.

## Methods

**Figure 4:**
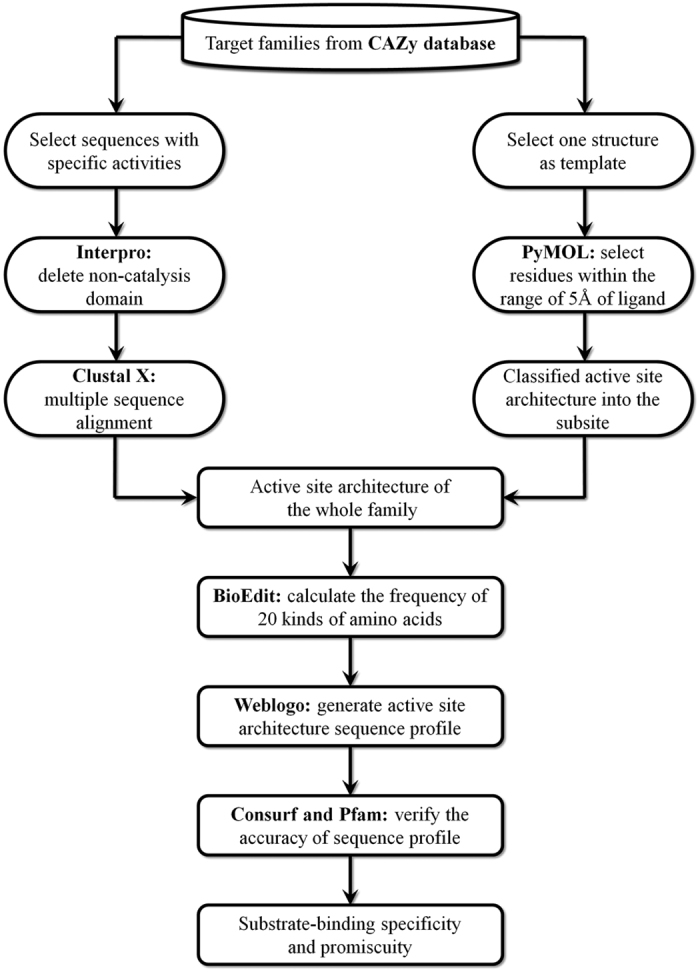
Flowchart of the active-site architecture analysis. Softwares and databases are in bold text.

A flowchart of the active-site architecture analysis is shown in Fig. 4.

### Data selection

Information on the cellulase, xylanase and β-glucosidase families was obtained from the glycoside hydrolase category of the CAZy database. For enzymes with the same activity that might distribute across more than one family, here we set a standard for screening the target family. First, the main enzyme activity of the target family must be one of the three selected activities; second, the entries listed at tab “characterized” that have specific activities in the target family must be larger than 50; third, there must be at least one available structure with corresponding enzyme activity and ligand as a template. For example, β-glucosidase (EC 3.2.1.21) is the main activity of the GH1 family, the total number of sequences with specific activities was 331 (by October 2015) and the structure “3F5J” could be used as a template, all meeting the standard above, so we selected the GH1 family as the target family. According to the standard, from all of the enzyme families containing the desired activity, we selected GH5, 6, 7, 9 and 12 as the target families of cellulase, GH10 and GH11 to represent xylanase and GH1 and GH3 to represent β-glucosidase (Supplementary Table S1). All sequences with specific activities and template structures of the above nine families were downloaded from NCBI database (GenBank)^55^ and Protein Data Bank^56^, respectively.

### Multiple sequence alignment

After obtaining all of the “characterized” sequences of a family, Interpro^57^ was used to detect and delete any non-catalysis domains, such as CBM and linker, for each sequence to update the original file. Then multiple sequence alignment was performed with ClustalX (gap open = 10.0, gap extend = 0.2)^58^. The whole process was applied to all nine families.

### Determination of amino acid residues making up the active site architecture

One structure with the ligands having the highest number of subsites and the corresponding enzyme activity was selected as the template. Amino acids within a range of 5 Å of the ligand were selected as the reference active site architecture by PyMOL (http://www.pymol.org/) and then recorded. The location of glycosidic bond cleavage was labeled as the cleavage site, from which subsites towards the reducing end were numbered +1, +2, … and those in the opposite direction were numbered −1, −2, …^41^,^59^. The selected active site architecture was further classified according to the subsite with which they had the shortest binding distance. Based on the results of multiple sequence alignment, we obtained the active site architecture of the whole family.

### Calculation of amino acid frequency

BioEdit (http://www.mbio.ncsu.edu/bioedit/bioedit.html) was used to calculate the frequency of 20 amino acids appearing in the active site architecture and the whole protein. And the PEARSON correlation coefficient^60^ (r) was used to represent the relevance between the two sets of data.

### Creation and testing of the sequence profile

The data constructed in the previous steps underwent format adjustments and was submitted to Weblogo^61^,^62^ to generate sequence profiles of active site architectures. In order to verify the accuracy of the sequence profiles, the scores for each column were calculated by the formula used by Weblogo^62^ and compared with the conservation scores from Consurf Sever^63^ and Jalview^64^.

## Additional Information

**How to cite this article**: Tian, L. *et al.* Ligand-binding specificity and promiscuity of the main lignocellulolytic enzyme families as revealed by active-site architecture analysis. *Sci. Rep.* **6**, 23605; doi: 10.1038/srep23605 (2016).

## Supplementary Material

Supplementary Information

## Figures and Tables

**Table 1:** The number of amino acid residues at potential subsites in cellulase families.

GH family	The number of amino acid residues at each subsite											Average number for each family	Average number for all families
	−7	−6	−5	−4	−3	−2	−1	+1	+2	+3	+4		
GH5	–	–	–	–	4	6	5	3	1	–	–	3.8	4.4
GH6	–	–	–	–	2	9	5	5	6	4	4	5	
GH7	3	5	5	6	6	6	3	9	5	–	–	5.3	
GH9	–	–	–	5	3	6	5	6	1	–	–	4.3	
GH12	–	–	–	2	3	3	5	7	3	–	–	3.8	
Average number for common subsites	–	–	–	–	3.6	6	4.6	6	3.2	–	–	–	–

In the table (similar to Tables 2, 3), the numbers of sequences analyzed in each family have been given in the beginning of the Results and Discussion. The number of amino acid residues at each ligand binding subsite for each enzyme family were counted, and the average amino acid residue number for each family as well as the average number for all families were further calculated. Due to the differences in ligand lengths, the average amino acid residue number at each common subsite of the ligand is also listed in the last row of table.

**Table 2:** The number of amino acid residues at potential subsites in xylanase families.

GH family	The number of amino acid residues at each subsite							Average number for each family	Average number for all families
	−3	−2	−1	+1	+2	+3	+4		
GH10	1	5	7	3	1	1	3	3	3.4
GH11	3	6	4	5	2	2	–	3.7	
Average number for common subsites	2	5.5	5.5	4	1.5	1.5	–	–	–

**Table 3:** The number of amino acid residues at potential subsites in β-glucosidase families.

GH family	The number of amino acid residues at each subsite		Average number for each family	Average number for all families
	−1	+1		
GH1	11	3	7	7.0
GH3	12	2	7	
Average number for common subsites	11.5	2.5	–	–
