# Supplementary material for: Ligand-binding specificity and promiscuity of the main lignocellulolytic enzyme families as revealed by active-site architecture analysis
Source: Sci Rep. 2016 Mar 24;6:23605. doi: 10.1038/srep23605 (PMC4806347; doi:10.1038/srep23605)
Supplement: Supplementary Information [file srep23605-s1.pdf]

Supplementary Information

Ligand-binding specificity and promiscuity of the main lignocellulolytic enzyme families as revealed by active-site architecture analysis

Li Tian[1], Shijia Liu[2], Shuai Wang[1], Lushan Wang[1,*]

[1]The State Key Laboratory of Microbial Technology, Shandong University, Jinan, 250100, P. R. China.

[2]Taishan College, Shandong University, Jinan, 250100, P. R. China.

*Corresponding author email: lswang@sdu.edu.cn

Figure legends

Figure S1. The correlation between number of potential subsites and length of tunnel or cleft.

Figure S2. The sequence profile of cellulase families.

Figure S3. The sequence profile of xylanase families.

Figure S4. The sequence profile of β-glucosidase families.

Figure S5. The interaction modes between conserved amino acid residues at -2 to +1 subsites and ligand in selected families.

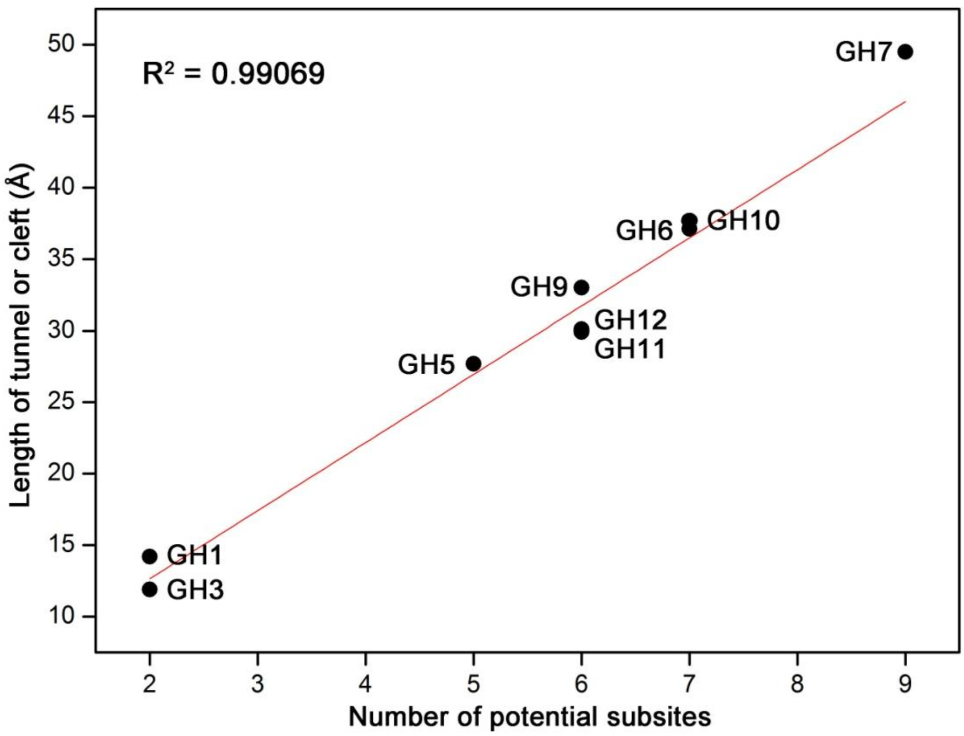

Figure S1. The correlation between number of potential subsites and length of tunnel or cleft. $R^2>0.99$ indicated that there is a linear dependency between number of potential subsites and length of tunnel or cleft.

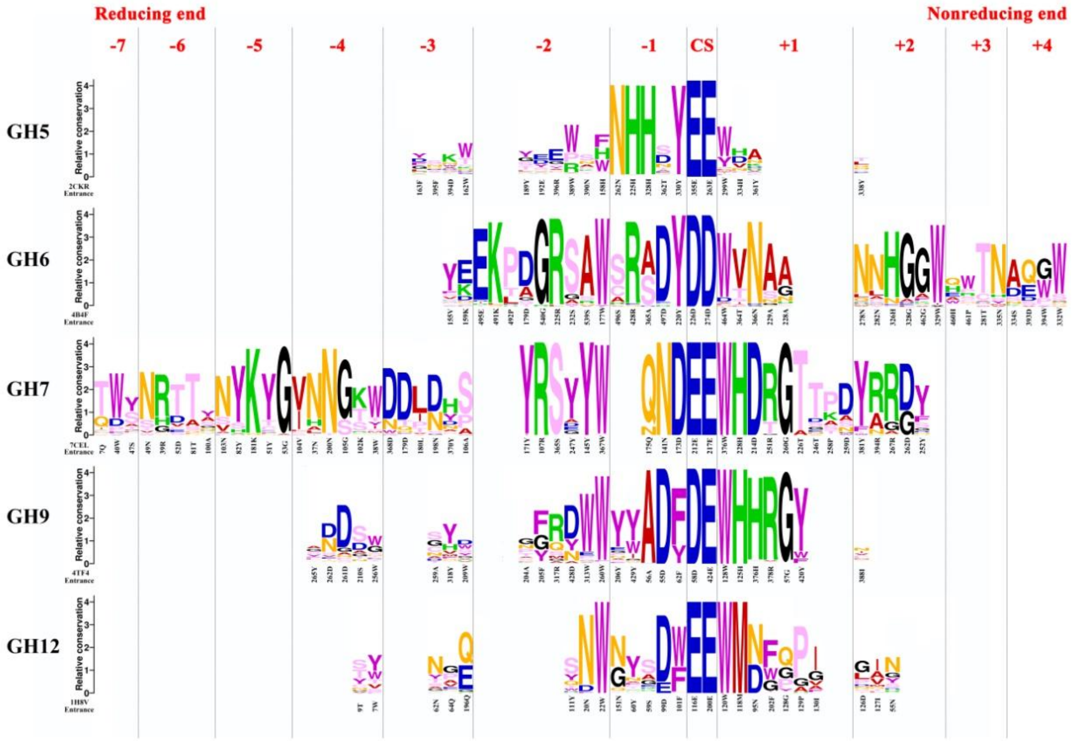

Figure S2. The sequence profile of cellulase families. The number of sequences that were aligned to create the sequence profile in GH5, GH6, GH7, GH9 and GH12 is 522, 63, 82, 157 and 65, respectively (all data above is valid to October 2015). All the sequences were characterized as specific activities.

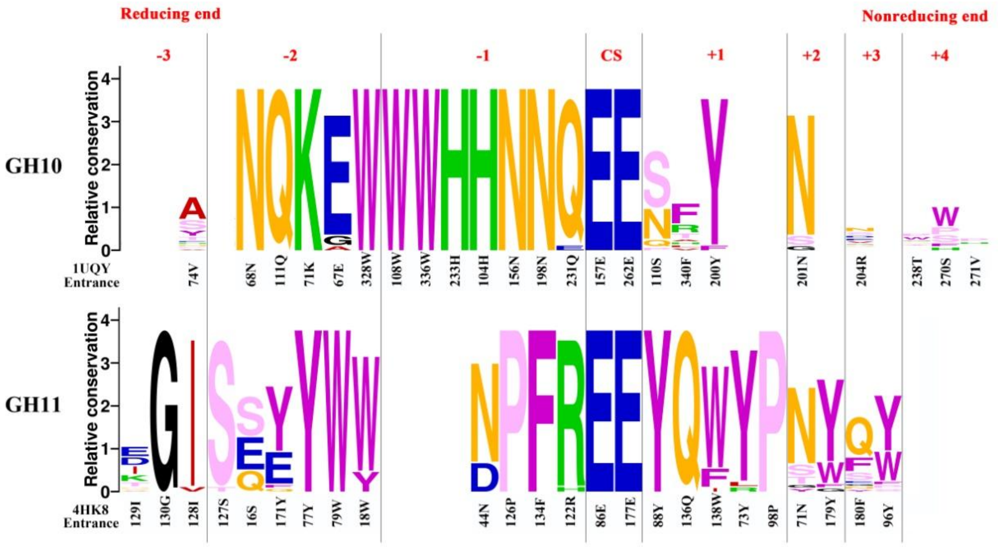

Figure S3. The sequence profile of xylanase families. 335 sequences were adopted to create profile in GH10, and 267 sequences were adopted in GH11 (all data above is valid to October 2015). All sequences were characterized as specific activities.

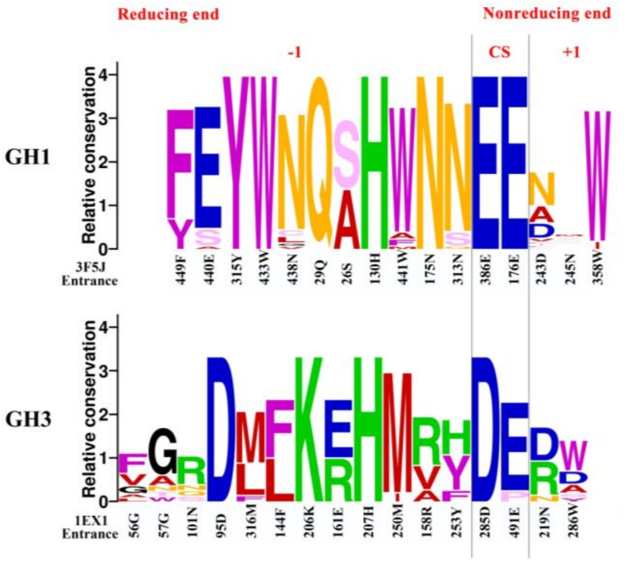

Figure S4. The sequence profile of β-glucosidase families. 331 sequences were adopted to create profile in GH1, and 278 sequences were adopted in GH3 (all data above is valid to October 2015). All the sequences were characterized as specific activities.

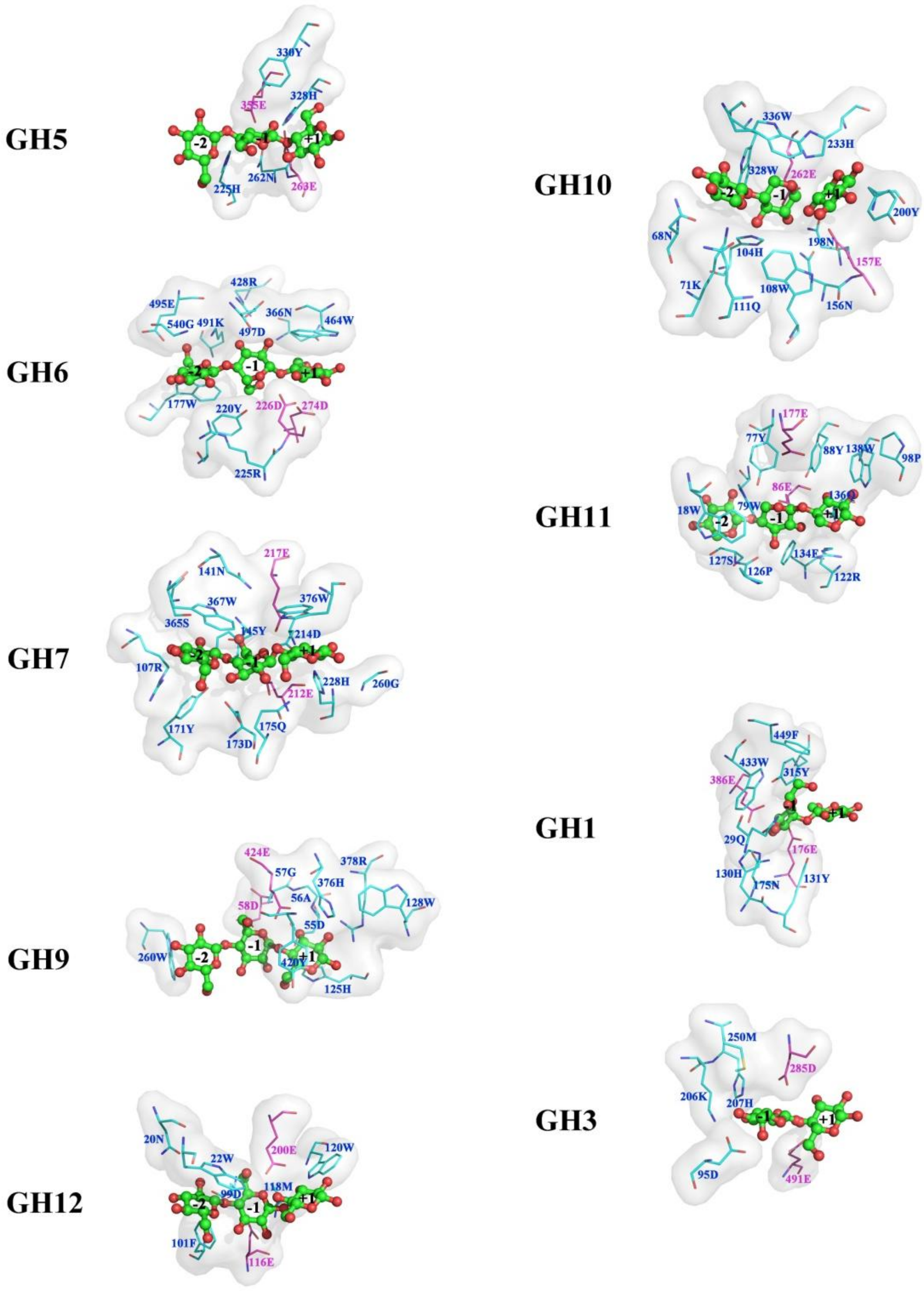

Figure S5. The interaction modes between conserved amino acid residues at -2 to +1 subsites and ligand in selected families. The space position of ligands and amino acid residues are displayed. Ligands are shown as sphere, with carbon atoms shown in green and oxygen atoms shown in red. Conserved amino acid residues are shown as sticks with cyan, except for two catalytic residues, which colored with magenta. All figures were prepared with PyMol.

Table legends

Table S1. The selection of target family.

Table S2. The major characteristics of nine lignocellulolytic enzyme families.

Table S3. The comparsion of the scores obtained from three methods.

Table S1. The selection of target family.

Enzyme	Family[a]	EC[b]	Sequence Number[c]	Template[d]
**Cellulase (EC#3.2.1.4/ 91/176)**	***GH5***	***3.2.1.4***	***522***	***2CKR***
	GH6	***3.2.1.4***	***63***	***4B4F***
	GH7	***3.2.1.176***	***82***	***7CEL***
	GH8	3.2.1.132	69	
	GH9	***3.2.1.4***	***157***	***4TF4***
	GH10	3.2.1.8	335	
	GH12	***3.2.1.4***	***65***	***1H8V***
	GH26	3.2.1.78	61	
	**GH44**	**3.2.1.4**	**15**	
	**GH45**	**3.2.1.4**	**47**	
	**GH48**	**3.2.1.176**	**15**	
	GH51	***3.2.1.4***	***73***	–
	**GH74**	**3.2.1.4**	**15**	
	**GH124**	**3.2.1.4**	**1**	
**Xylanase (EC#3.2.1.8)**	GH5	3.2.1.4	522	
	GH8	3.2.1.132	69	
	GH9	3.2.1.4	157	
	GH10	***3.2.1.8***	***335***	***1UQY***
	GH11	***3.2.1.8***	***267***	***4HK8***
	GH12	3.2.1.4	65	
	GH16	2.4.1.207	197	
	GH26	3.2.1.78	61	
	**GH30**	**3.2.1.8**	**32**	
	GH43	3.2.1.37	131	
	GH44	3.2.1.4	15	
	GH51	3.2.1.4	73	
	GH62	3.2.1.55	21	
	GH98	3.2.1.102	5	
**β-glucosidase (EC#3.2.1.21)**	***GH1***	***3.2.1.21***	***331***	***3F5J***
	GH2	3.2.1.23	142	
	GH3	***3.2.1.21***	***278***	***1EX1***
	GH5	3.2.1.4	522	
	GH9	3.2.1.4	157	
	GH30	3.2.1.8	32	
	**GH116**	**3.2.1.21**	**5**	

[a]According to the information obtained from CAZy database (http://www.cazy.org/Glycoside-Hydrolases.html), cellulase, xylanase and β-glucosidase are distributed in the above families.

[b,c,d]Here, we set a standard for screening the target family. First, the main enzyme activity of the target family must be one of the three selected activities, eligible family shown in **bold**; second, the entries listed at tab "characterized" that have specific activities in the target family must be larger than 50;, family satisfies the above two criterias shown in ***bold and italic***; third, there must be at least one available structure with corresponding enzyme activity and ligand as a template, family meeting all conditions shown in ***bold and italic***. Finally, we selected GH5, 6, 7, 9 and 12 as the target families of cellulase, GH10 and GH11 to represent xylanase and GH1 and GH3 to represent β-glucosidase (the all data above are only valid to October 2015).

Table S2. The major characteristics of nine lignocellulolytic enzyme families[a].

Enzyme	GH family	Clan	3D structure status	Catalytic mechanism	Catalytic nucleophile/base	Catalytic proton donor	Distance between catalytic residues[b]	Length of tunnel or cleft[b]	Number of potential subsites
**Cellulase**	GH5	GH-A	$(\beta/\alpha)_8$	Retaining	Glu	Glu	5.1Å	27.7Å	5
	GH6	-	-	Inverting	Asp	Asp	10.2Å	37.1Å	7
	GH7	GH-B	β-jell roll	Retaining	Glu	Glu	5.1Å	49.5Å	9
	GH9	-	$(\alpha/\alpha)_6$	Inverting	Glu	Asp	7.2Å	33.0Å	6
	GH12	GH-C	β-jell roll	Retaining	Glu	Glu	5.3Å	30.1Å	6
**Xylanase**	GH10	GH-A	$(\beta/\alpha)_8$	Retaining	Glu	Glu	5.5Å	37.7Å	7
	GH11	GH-C	β-jell roll	Retaining	Glu	Glu	5.1Å	29.9Å	6
**β-glucosidase**	GH1	GH-A	$(\beta/\alpha)_8$	Retaining	Glu	Glu	5.2Å	14.2Å	2
	GH3	-	-	Retaining	Asp	Glu (partly)	6.2Å	11.9Å	2

[a]The information of nine lignocellulolytic enzyme families obtained from CAZy database[1].

[b]The distance or length was measured by PyMOL (http://www.pymol.org/).

Table S3. The comparsion of the scores obtained from three methods.

A. The scores of conserved amino acid residues in GH5.

Conserved residues[a]	262N	225H	330Y	355E	263E	328H
**Weblogo[b]**	4.079	4.097	4.088	4.322	4.322	4.088
**Consurf[c]**	9	9	9	9	9	9
**Jalview[d]**	2	10	0	10	10	10

B. The scores of conserved amino acid residues in GH6.

Conserved residues	491K	540G	225R	177W	428R	220Y	497D	226D	274D	366N
**Weblogo**	3.741	4.030	4.030	4.030	4.030	4.176	3.741	4.322	4.322	4.030
**Consurf**	9	9	9	9	9	9	9	9	9	9
**Jalview**	4	10	10	10	10	10	10	10	10	10

C. The scores of conserved amino acid residues in GH7.

Conserved residues	107R	365S	171Y	367W	173D	175Q	141N	145Y	214D	212E	217E	376W	228H	260G
**Weblogo**	4.322	4.322	4.204	4.204	4.322	3.819	4.322	4.322	4.322	4.322	4.322	4.322	4.322	4.322
**Consurf**	9	9	9	9	9	9	9	9	9	9	9	9	9	9
**Jalview**	11	11	10	10	11	9	11	11	11	10	10	11	11	11

D. The scores of conserved amino acid residues in GH9.

Conserved residues	260W	62F	56A	55D	58D	424E	420Y	125H	376H	378R	57G	128W
**Weblogo**	3.923	3.458	4.252	4.322	4.322	4.322	3.469	2.467	3.923	3.443	4.183	2.467
**Consurf**	8	9	9	9	9	9	8	9	9	9	9	9
**Jalview**	11	9	10	11	10	10	9	10	10	9	10	10

E. The scores of conserved amino acid residues in GH12.

Conserved residues	20N	22W	101F	99D	116E	200E	120W	118M
**Weblogo**	3.622	4.176	3.236	3.723	4.322	4.322	4.322	4.322
**Consurf**	9	9	8	9	9	9	9	9
**Jalview**	8	11	9	9	11	11	11	11

F. The scores of conserved amino acid residues in GH10.

Conserved residues	68N	111Q	71K	328W	336W	233H	104H	156N	198N	231Q	157E	262E	108W	200Y
**Weblogo**	4.322	4.322	4.322	4.322	4.322	4.322	4.322	4.322	4.322	4.087	4.322	4.322	4.322	4.087
**Consurf**	9	9	9	9	9	9	9	9	9	9	9	9	9	9
**Jalview**	11	11	11	11	11	11	11	11	11	8	11	11	11	9

G. The scores of conserved amino acid residues in GH11.

Conserved residues	130G	128I	127S	77Y	79W	18W	126P	134F	122R	86E	177E	88Y	136Q	98P
**Weblogo**	4.322	4.087	4.087	4.322	4.322	3.703	4.322	4.322	4.087	4.322	4.322	4.322	4.322	4.087
**Consurf**	9	9	9	9	9	8	9	9	9	9	9	9	9	9
**Jalview**	11	9	9	11	11	8	11	11	8	11	11	11	11	8

H. The scores of conserved amino acid residues in GH1.

Conserved residues	449F	315Y	433W	29Q	130H	175N	386E	176E
**Weblogo**	3.563	4.322	4.322	4.322	4.322	4.322	4.322	4.322
**Consurf**	8	9	9	9	9	9	9	9
**Jalview**	9	11	11	11	11	11	11	11

I. The scores of conserved amino acid residues in GH3.

Conserved residues	95D	206K	207H	250M	585D
**Weblogo**	4.322	4.322	4.322	3.951	4.322
**Consurf**	9	9	9	9	9
**Jalview**	11	11	11	10	11

[a]If residues of the same type, or with similar properties, make up more than 90% of the sequences in any column of multiple sequence alignment, the residues on this column is defined as conserved.

[b]The score obtained from Weblogo[2] is range from 0 to 4.322, the residue with higher score is more conserved.

[c] The score obtained from Consurf[3] is range from 1 to 9, the residue with higher score is more conserved.

[d] The score obtained from Jalview[4] is range from 1 to11, the residue with higher score is more conserved.

Reference

1 Lombard, V., Golaconda Ramulu, H., Drula, E., Coutinho, P. M. & Henrissat, B. The carbohydrate-active enzymes database (CAZy) in 2013. *Nucleic Acids Res*. **42**, D490-D495 (2013).

2 Crooks, G. E., Hon, G., Chandonia, J. M. & Brenner, S. E. WebLogo: a sequence logo generator. *Genome Res*. **14**, 1188-1190 (2004).

3 Ashkenazy, H., Erez, E., Martz, E., Pupko, T. & Ben-Tal, N. ConSurf 2010: calculating evolutionary conservation in sequence and structure of proteins and nucleic acids. *Nucleic Acids Res.* **38**, W529-533 (2010).

4 Waterhouse, A. M., Procter, J. B., Martin, D. M., Clamp, M. & Barton, G. J. Jalview Version 2--a multiple sequence alignment editor and analysis workbench. *Bioinformatics.* **25**, 1189-1191 (2009).
